# Plasma metabolite octadecadienedioate: A novel protective factor against diabetes-related Charcot foot

**DOI:** 10.1097/MD.0000000000049973

**Published:** 2026-07-31

**Authors:** Daosen Zhou, Diya Xie, Lihang Yang, Cheng Li, Fengmin Liu

**Affiliations:** a Department of General Surgery, Fuzhou First General Hospital Affiliated with Fujian Medical University, Fuzhou, Fujian, China; b Department of Endocrinology, Fuzhou First General Hospital Affiliated with Fujian Medical University, Fuzhou, Fujian, China.

**Keywords:** diabetes-related Charcot foot, Mendelian randomization, plasma metabolites

## Abstract

Charcot foot (CF) is a severe, progressive neuro-osteoarthropathy predominantly affecting diabetic patients with peripheral neuropathy. Characterized by progressive bone and joint destruction, CF can lead to debilitating foot deformities, ulceration, infection, and ultimately lower extremity amputation. Despite its significant clinical impact, the global prevalence remains uncertain, with an estimated 1.6 million people affected worldwide. Early diagnosis is challenging due to the insidious onset and limited specificity of current clinical, imaging, and laboratory assessments. The role of circulating metabolites in CF pathogenesis remains poorly understood. This study aimed to investigate the causal relationship between plasma metabolites and CF risk using Mendelian randomization (MR) and to identify potential biomarkers for early detection and therapeutic intervention. We conducted a bidirectional 2-sample MR study using genome-wide association study summary data. Plasma metabolite data were obtained from a cohort of approximately 8000 individuals of European descent (genome-wide association study catalog: GCST90199621–GCST90201020), and CF summary statistics were derived from the FinnGen Study (9th version), comprising 473 cases and 271,817 controls. Genetic instrumental variables were selected through linkage disequilibrium clumping and harmonization. The inverse-variance weighted method served as the primary analysis, supplemented by MR-Egger, weighted median, and weighted mode methods. Sensitivity analyses included Cochran *Q* test, Egger intercept test, MR-Pleiotropy RESidual Sum and Outlier, and leave-one-out analyses. Multivariable MR and colocalization analyses were performed to validate the findings. After false discovery rate adjustment, octadecadienedioate demonstrated a significant negative association with CF risk (odds ratio = 0.644, 95% confidence interval 0.528–0.785, false discovery rate = 0.018). This protective association was consistent across all MR methods, robust in sensitivity analyses, and further supported by multivariable MR (odds ratio = 0.632, 95% confidence interval 0.479–0.834) and colocalization analysis (posterior probability 4 = 0.945). Reverse MR analysis revealed no significant causal effects from CF to metabolite levels. Our findings identify octadecadienedioate as a novel protective factor against CF, highlighting the potential of circulating metabolites in understanding CF pathogenesis. These results may inform future preventive strategies, biomarker development, and personalized diabetic foot care.

## 1. Introduction

Diabetic foot disease, a long-acknowledged primary cause of global amputations, continues to pose significant health challenges.^[1]^ A 2023 review indicates that minor amputation (defined as amputations distal to the ankle, including toe and partial foot amputations) rates among diabetic individuals have risen from 1.2 to 1.4 per 1000 person-years in the 21st century.^[2]^ Consequently, the 2023 International Working Group on the Diabetic Foot updated its definition of diabetic foot disease to encompass peripheral neuropathy, peripheral artery disease, infections, ulcers, neuro-osteoarthropathy, gangrene, and amputation in individuals with diabetes.^[3]^

Jean-Martin Charcot significantly advanced our comprehension of Charcot foot (CF), particularly its association with the central nervous system in diabetes patients.^[4]^ CF, predominantly observed in diabetic individuals, affects the foot and ankle but can manifest in anyone suffering from peripheral neuropathy. The actual incidence and prevalence of CF in diabetes remain uncertain, largely due to the neuropathy-induced lack of pain delaying medical consultations. Lavery et al noted an annual incidence of 0.85%, with non-Hispanic whites exhibiting higher CF rates than Mexican Americans.^[5]^ According to the International Diabetes Foundation, an estimated 537 million adults were living with diabetes in 2021. Assuming a prevalence of 0.3%, it’s estimated that around 1.6 million people globally live with CF, with an annual incidence of 160,000 new cases.^[6]^ Delayed intervention can lead to foot and ankle deformations, escalating the risk of ulcers, infections, and amputations. Regrettably, the diagnosis of CF can be obscured due to the limited specificity of physical examinations, imaging, and laboratory results.

The pathophysiology of CF involves neurotraumatic, inflammatory, and metabolic components. Peripheral neuropathy abolishes protective sensation, enabling repetitive microtrauma that triggers localized inflammation and osteoclast-driven bone resorption. Chronic hyperglycemia exacerbates this process through AGE-mediated nuclear factor-kappa B activation, fostering a sustained inflammatory and oxidative milieu that compromises skeletal integrity.^[7]^ Established risk factors include diabetes duration exceeding 10 years, HbA1c levels above 6.5 to 7%, peripheral neuropathy, renal impairment, obesity, and prior foot ulceration.^[8,9]^ Notably, diabetes duration ≥ 10 years confers the strongest risk (odds ratio [OR] 6.7, 95% confidence interval [CI] 2.0–21.6), while HbA1c > 6.5% is an independent predictor of CF development.^[9,10]^ Clinically, CF differs from other diabetic foot complications such as diabetic foot ulcer (DFU), which results from combined neuropathy, vascular insufficiency, and external trauma. CF, by contrast, presents as a warm, swollen, erythematous foot deformity without primary vascular compromise; indeed, peripheral arterial disease may exert a protective effect by attenuating regional hyperemia and osteoclastic activity.^[7]^ This nonspecific presentation often mimics infection or deep vein thrombosis, contributing to diagnostic delay.

Furthermore, current diagnostic modalities face substantial limitations that impede early recognition. Physical examination findings (such as unilateral warmth, erythema, and edema) lack specificity, as these features overlap with soft tissue infection, gout, or deep vein thrombosis.^[11]^ Conventional radiography, often the first-line imaging modality, exhibits very poor sensitivity in the early stages of CF and typically fails to detect bone marrow edema for up to 4 weeks after disease onset.^[11]^ Magnetic resonance imaging, while offering high sensitivity for bone marrow edema, cannot reliably differentiate sterile neuroinflammatory changes from osteomyelitis, frequently yielding false-positive results when bone marrow edema is used as the sole diagnostic criterion.^[12]^ Nuclear medicine techniques, including radiolabeled white blood cell scintigraphy and fluorine-18 fluorodeoxyglucose positron emission tomography/computed tomography, also demonstrate limited specificity in the mid- and hindfoot, where chronic bone marrow expansion secondary to repetitive microtrauma may mimic infectious involvement.^[13]^ Consequently, up to 25% of CF cases are reportedly misdiagnosed at initial presentation, with patients often undergoing unnecessary antibiotic therapy or surgical intervention for presumed infection.^[14]^ These diagnostic challenges underscore the urgent need for novel, objective biomarkers capable of early risk stratification and timely identification of individuals at the highest risk for CF development.

Circulating metabolites, small molecular entities originating from tissues, biological fluids, and cells, span a broad spectrum of compounds like xenobiotics, lipids, amino acids, and carbohydrates.^[15]^ The high sensitivity of metabolomics allows for the detection of subtle shifts in biological behavior, providing insight into various physiological states, abnormal processes, and diseases.^[16]^ In the specific context of diabetic foot complications, recent metabolomic investigations have identified distinct metabolic signatures. Wang et al demonstrated that lower serum branched-chain amino acid catabolic intermediates (including 3-methyl-2-oxovaleric acid, succinic acid, and methylmalonic acid) represent predictive signatures specific to patients with diabetic foot compared to those with uncomplicated type 2 diabetes mellitus, suggesting that metabolic dysregulation may precede and contribute to the onset of diabetic foot complications.^[17]^ However, no study to date has specifically investigated the association between circulating metabolites and CF risk, leaving a critical gap in our understanding of the metabolic determinants of this devastating complication.

Traditional statistical methods used to infer causality from observational data are prone to confounding bias. Mendelian randomization (MR) analysis is a technique that employs genetic variations as instrumental variables (IVs) to investigate the causal links between potential alterable exposures and health outcomes in observational research. This method, which is widely applicable, is built on the foundation of genome-wide association studies (GWAS) summary data.^[18]^ In other words, the genetic variations identified in GWAS can act as proxies for a randomized controlled trial, thereby establishing a causal linkage between various factors.^[19]^ Prior studies have predominantly used a bidirectional MR approach to focus on metabolites that affect diabetes, but these studies have been limited to certain complications of diabetes.^[20]^ Nevertheless, there is currently no investigation on the correlation between metabolites and CF using larger sample data.

Consequently, to address this gap, we leveraged publicly available large-scale GWAS summary data (including plasma metabolite data from the GWAS catalog [GCST90199621 to GCST90201020, approximately 8000 European individuals] and CF data from the FinnGen Study [9th version, 473 cases and 271,817 controls]) to conduct a bidirectional MR analysis to probe the causal relationship between blood metabolites and CF. Furthermore, it aimed to probe deeper into their role in common complications of type 2 diabetes. This could offer fresh insights into future preventative measures, early intervention strategies, and clinical management of CF.

## 2. Study design

It is important to clarify that this 2-sample MR (TSMR) study relied exclusively on publicly available GWAS summary statistics, not on individual-level data. Consequently, no actual plasma metabolite concentrations were measured in our laboratory; rather, we used preexisting genetic variant–metabolite association summary data (beta coefficients, standard errors, and effect allele frequencies) from the GWAS catalog.

**Figure 1.**
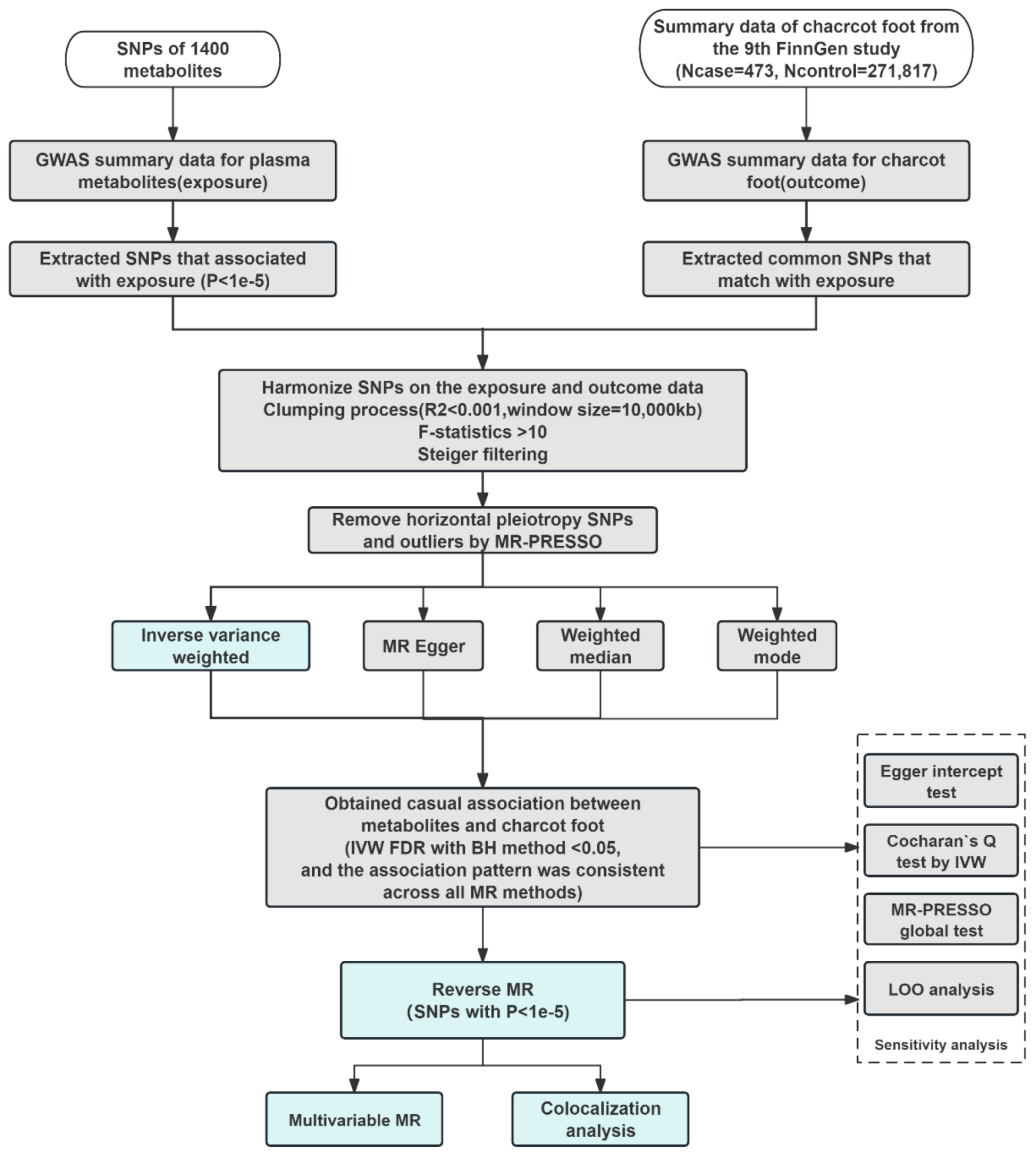
Bidirectional TSMR study design. Instruments selected at *P* < 1 × 10^−5^, LD *R*^2^ < 0.001 (10,000 kb), Steiger-filtered, *F* > 10. Primary: IVW; supplementary: MR-Egger, weighted median, weighted mode. Sensitivity: Cochran *Q*, Egger intercept, MR-PRESSO, LOO. Significance: FDR < 0.05 (BH). Validated by reverse MR, MVMR (adjusting for cigarette consumption, BMI, fasting glucose), and colocalization. BMI = body mass index, BH = Benjamini–Hochberg, FDR = false discovery rate, IVW = inverse-variance weighted, LD = liquid disequilibrium, LOO = leave-one-out, MR = Mendelian randomization, MR-PRESSO = MR-Pleiotropy RESidual Sum and Outlier, MVMR = multivariable Mendelian randomization, TSMR = 2-sample Mendelian randomization.

The study design centered on plasma metabolite levels as exposures and CF as the outcome, as shown in Figure 1. Rigorous selection of genetic IVs was performed using linkage disequilibrium (LD) clumping and harmonization of effect alleles. For the robustness of an MR study, each IV needs to have a significant correlation with the exposure, be free from confounding influences, and affect the outcome exclusively via the exposure. We applied the inverse-variance weighted (IVW) method as the primary approach for MR analysis, with additional methods (MR-Egger, weighted mode, and weighted median) for verification. We performed sensitivity analyses, incorporating Cochran *Q* test for heterogeneity, Egger intercept test and MR-Pleiotropy RESidual Sum and Outlier (MR-PRESSO) global test for pleiotropy and outlier single nucleotide polymorphisms (SNPs), and leave-one-out (LOO) analyses for identifying dominant single SNPs. Reverse MR analyses were performed to examine reverse causality, and power calculations were conducted for statistical strength assessment. Multivariable MR (MVMR) analyses adjusted for confounders, and colocalization analysis explored shared genetic foundations between plasma metabolites and CF risk. Data analysis was conducted using R software (V.4.2.3; R Foundation for Statistical Computing), with a *P* of < .05 for all sensitivity analyses.

### 2.1. Data sources

Table S1, Supplemental Digital Content 1 provides an overview of the data sources. This TSMR study relied exclusively on publicly available GWAS summary statistics, not on individual-level data. Plasma metabolite data (accession numbers GCST90199621 to GCST90201020) were obtained from a cohort of approximately 8000 individuals of European descent,^[21]^ providing genetic variant–metabolite association summary statistics (beta coefficients, standard errors, and effect allele frequencies) from the GWAS catalog (https://www.ebi.ac.uk/gwas/). This cohort comprised a general population sample with a diabetes prevalence of approximately 15%, and the GWAS summary statistics reflect genetic associations with metabolite levels across diabetic and nondiabetic participants. CF summary data were derived from the 9th version of the FinnGen Study, comprising 473 cases and 271,817 controls, as detailed in a previous study.^[22]^ The CF endpoint was defined by International Classification of Diseases-10 codes M14.6E10.6 and M14.6E11.6, which specifically capture diabetic Charcot neuroarthropathy; therefore, all CF cases were diabetic patients. The metabolite GWAS cohort and the FinnGen CF cohort are completely independent, with no participant overlap. Summary statistics of factors previously established to be associated with DFU were obtained from the Integrative Epidemiology Unit open GWAS project (https://gwas.mrcieu.ac.uk/).

### 2.2. Filtering of genetic IVs

LD clumping was conducted with a 10,000 kilobase window size to select SNPs independently associated with plasma metabolites (pairwise LD *R*^2^ < 0.001) at *P* < 1 × 10^−5^. Palindromic SNPs with median allele frequencies were eliminated. To lessen the effect of weak instruments, SNPs featuring *F*-statistics < 10 were excluded. Using the formula below, *F*-statistics are determined with, *R*^2^ denoting genetic variation, N representing the sample size, *β* signifying the allele effect value, and EAF indicating the effect allele frequency.


$$F=R^{2}\frac{\left(N-2\right)}{\left(1-R^{2}\right)}$$

$$\begin{array}{c} R^{2}=2\times EAF\times \left(1-EAF\right)\times \beta^{2} \end{array}$$

For the reverse MR, the process of choosing IVs was consistent with the previously mentioned steps, employing genome-wide significance (*P* < 1 × 10^−5^) to select CF-associated SNPs, as no significant IVs remained with a *P* threshold set at < 5 × 10^−8^. Using the LD trait function of the LDlinkR package (v1.3.0), we investigated whether the CF IVs had been previously linked with any metabolites and found no reported associations.^[23]^

### 2.3. MR analysis

The IVW method was employed for the primary analysis. We used a random-effects model for the existence of heterogeneity (*P* < .05) and a fixed-effects model in its absence.^[24]^ To confirm the solidity of the results, we incorporated 3 supplementary MR approaches: MR-Egger, weighted median, and weighted mode. Specifically, the MR-Egger regression was used to detect and correct for pleiotropic effects arising from genetic variants influencing both the outcome and exposure. To counter potential bias from the IVW’s assumption of all IVs being valid, we applied the weighted median method, which considers that up to half of the IVs could be invalid.^[25]^ Moreover, MR-PRESSO was used to identify and correct for pleiotropy and outlier SNPs. To guarantee the strength of our findings, we implemented the Benjamini and Hochberg correction to achieve a false discovery rate (FDR). The statistical analyses were conducted using R packages MR-PRESSO (v1.0) and TwoSampleMR (v0.5.8).^[26]^

### 2.4. Sensitivity and reverse analyses

To ensure the reliability of associations, we utilized Cochran *Q* test with the IVW method to assess heterogeneity, performed the Egger intercept test and MR-PRESSO global test to investigate horizontal pleiotropy, and employed LOO analyses to identify dominant IVs.^[27]^ We further conducted reverse MR analyses, considering CF as the exposure and each of the 1400 metabolites served as the outcome to investigate potential reverse causal associations.

We considered an exposure-outcome association to be significantly reliable if it met the following criteria: the association’s significance reached an FDR < 0.05 in IVW and a *P* value < .05 in at least 1 of the remaining 3 methods; the association’s pattern was maintained consistent throughout all of the MR methods; each IVs used exhibited an *F*-values > 10; absence of notable heterogeneity was noted among the IVs; absence of horizontal pleiotropy was confirmed, with both an Egger *P* value for intercept and an MR-PRESSO global test *P* value exceeding .05; the MR estimates showed stability, with no single IV demonstrating dominance in the LOO analyses.

### 2.5. Power calculation

We employed a platform (https://shiny.cnsgenomics.com/mRnd/) for assessing the statistical power of MR estimates.^[28]^ This platform applies asymptotic theory to compute power values for identifying causal effects originating from IVs. The calculations were conducted at a type I error rate of 0.05, considering parameters including the *R*^2^ of the IVs, the proportion of CF cases in the dataset, and the OR derived from the IVW method in our analyses.

### 2.6. MVMR analysis

MVMR analyses were applied to assess the direct influence of metabolite levels on CF, independent of potential confounders. Factors related to DFU, including daily cigarette consumption, cigarette initiation, body mass index, fasting glucose, and years of schooling, were identified based on a previous study.^[29]^ We carried out TSMR analysis for each of these factors against CF (SNPs independently associated with exposures were selected at *P* < 1 × 10^−8^). However, cigarette initiation was omitted due to insufficient shared SNPs with the outcome even when *P* < 1 × 10^−5^. Significant exposures in TSMR were further investigated as confounders in MVMR analysis. MVMR effectively integrates multiple interacting exposures to address intricacies arising from interrelations among genetic variants linked to various exposures, with analyses executed using the R package MVMR (v0.4).^[30]^

### 2.7. Colocalization analysis

Colocalization analysis can enhance MR results by addressing its constraints associated with pleiotropy and LD, offering profound insight into the common genetic basis of exposures and outcomes.^[29]^ We evaluated the colocalization between the metabolite and CN risk at the genetic locus of each IV using the R package coloc (v5.2.3) to assess if the identified relationships were affected by high LD, as indicated in prior research.^[31]^ An indication of strong colocalization was inferred when the posterior probability of H4 exceeded 0.8.

## 3. Results

### 3.1. Selection of IVs

We acquired summary statistics for 28,197 common genetic variants (Table S2, Supplemental Digital Content 2) using Steiger filtering (*P* > .05). The primary MR analysis for these metabolites utilized a range of 11 to 82 IVs after excluding weak IVs with *F*-statistics < 10 and outliers (MR-PRESSO outlier test *P* < .05) (Table S3, Supplemental Digital Content 3). In the reverse analysis, the final count of filtered IVs for CF ranged from 2 to 10 (Table S4, Supplemental Digital Content 4).

### 3.2. Causal associations between metabolites and CF

Using the IVW method as the primary analysis (*P* < .05), and 3 other MR methods pointing in the same direction, we identified 60 metabolites potentially significantly associated with CF; 34 negatively and 26 positively (Table S5, Supplemental Digital Content 5). The explained variance in plasma metabolite levels by these IVs ranged from 1.530 to 60.964%.

**Figure 2.**
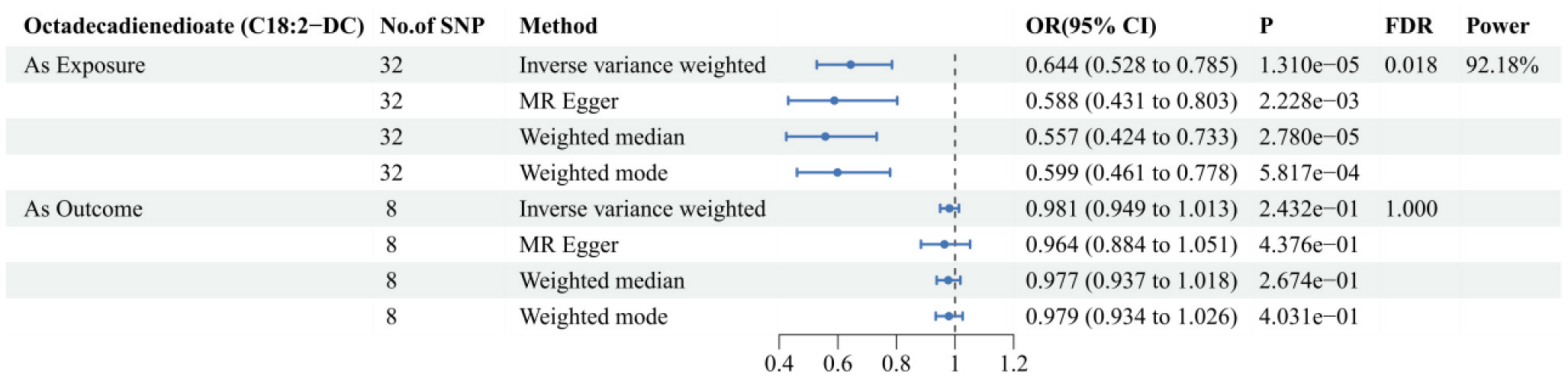
Forward and reverse MR estimates for C18:2-DC and CF. Forward (as exposure): 32 SNPs, C18:2-DC → CF. Reverse (as outcome): 8 SNPs, CF → C18:2-DC. ORs with 95% CIs shown for IVW, MR-Egger, weighted median, and weighted mode, with *P* values, FDR, and statistical power. C18:2-DC = octadecadienedioate, CF = Charcot foot, CI = confidence interval, FDR = false discovery rate, IVW = inverse-variance weighted, MR = Mendelian randomization, OR = odds ratio, SNP = single-nucleotide polymorphism.

**Figure 3.**
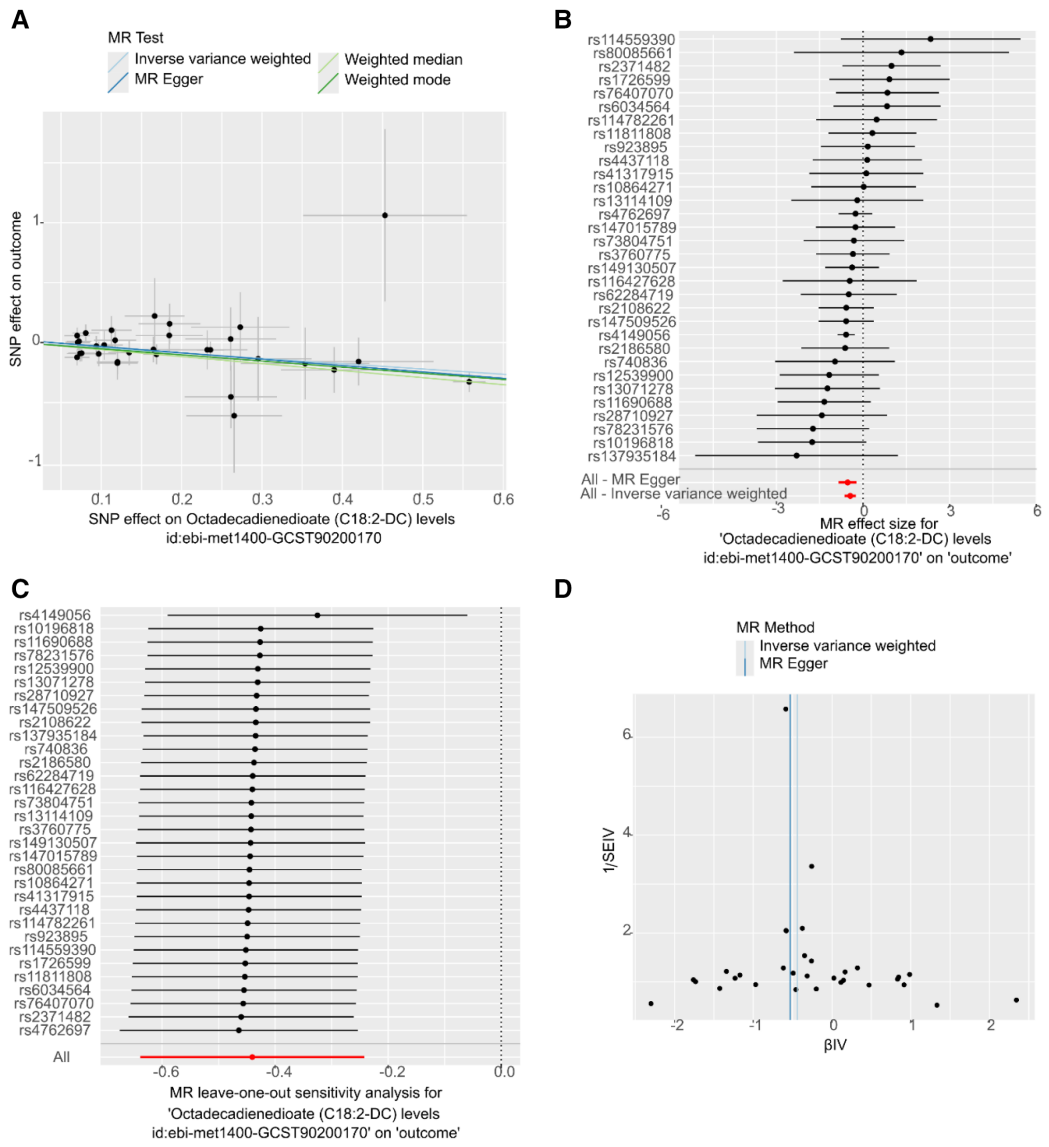
Sensitivity analyses for the causal effect of C18:2-DC on CF (32 SNP instruments). (A) Scatter plot: per-SNP effects on C18:2-DC vs CF; negative slopes confirm protective effect. (B) LOO analysis: IVW reestimated excluding each SNP sequentially. No single SNP drives the result. (C) Forest plot: per-SNP log OR with 95% CI; red diamonds = IVW and MR-Egger pooled estimates. (D) Funnel plot: symmetric distribution indicates no directional pleiotropy (Egger intercept *P* = .466; MR-PRESSO *P* = .771). C18:2-DC = octadecadienedioate, CF = Charcot foot, CI = confidence interval, IVW = inverse-variance weighted, LOO = leave-one-out, MR = Mendelian randomization, MR-PRESSO = MR-Pleiotropy RESidual Sum and Outlier, OR = odds ratio, SNP = single-nucleotide polymorphism.

After adjusting for an FDR value of < 0.05, we discerned a significant negative effect of a specific metabolite, octadecadienedioate (C18:2-DC), on CF risk with an OR of 0.644 (95% CI 0.528–0.785, FDR = 0.018) (Fig. 1 and 2, Table S5, Supplemental Digital Content 5). The same association patterns were observed by the other 3 MR methods (Fig. 2). LOO analyses verified that this association was not driven by a single IV (Fig. 2). Both Egger regression analysis (*P* = .466) and MR-PRESSO global tests (*P* = .771) suggested that horizontal pleiotropy didn’t impact the association (Fig. 3A–D). Furthermore, no significant heterogeneity was identified (*P* = .758) (Table S5, Supplemental Digital Content 5).

### 3.3. Reverse MR analysis

Reverse MR analysis was performed to explore the association between CF as the exposure and the levels of each of the 1400 plasma metabolites as outcomes (Fig. 1, Table S6, Supplemental Digital Content 6). We annotated association results using LDlinkR, and no SNP was excluded in this analysis (Table S7, Supplemental Digital Content 7). By applying the filtered IVs and the IVW method, with FDR < 0.05 as the significance threshold, we found no significant correlations between CF and any of the 1400 metabolite levels. Additionally, we assessed heterogeneity, pleiotropy, and conducted MR-PRESSO global tests in this reverse MR analysis.

### 3.4. MVMR and colocalization analysis

**Figure 4.**
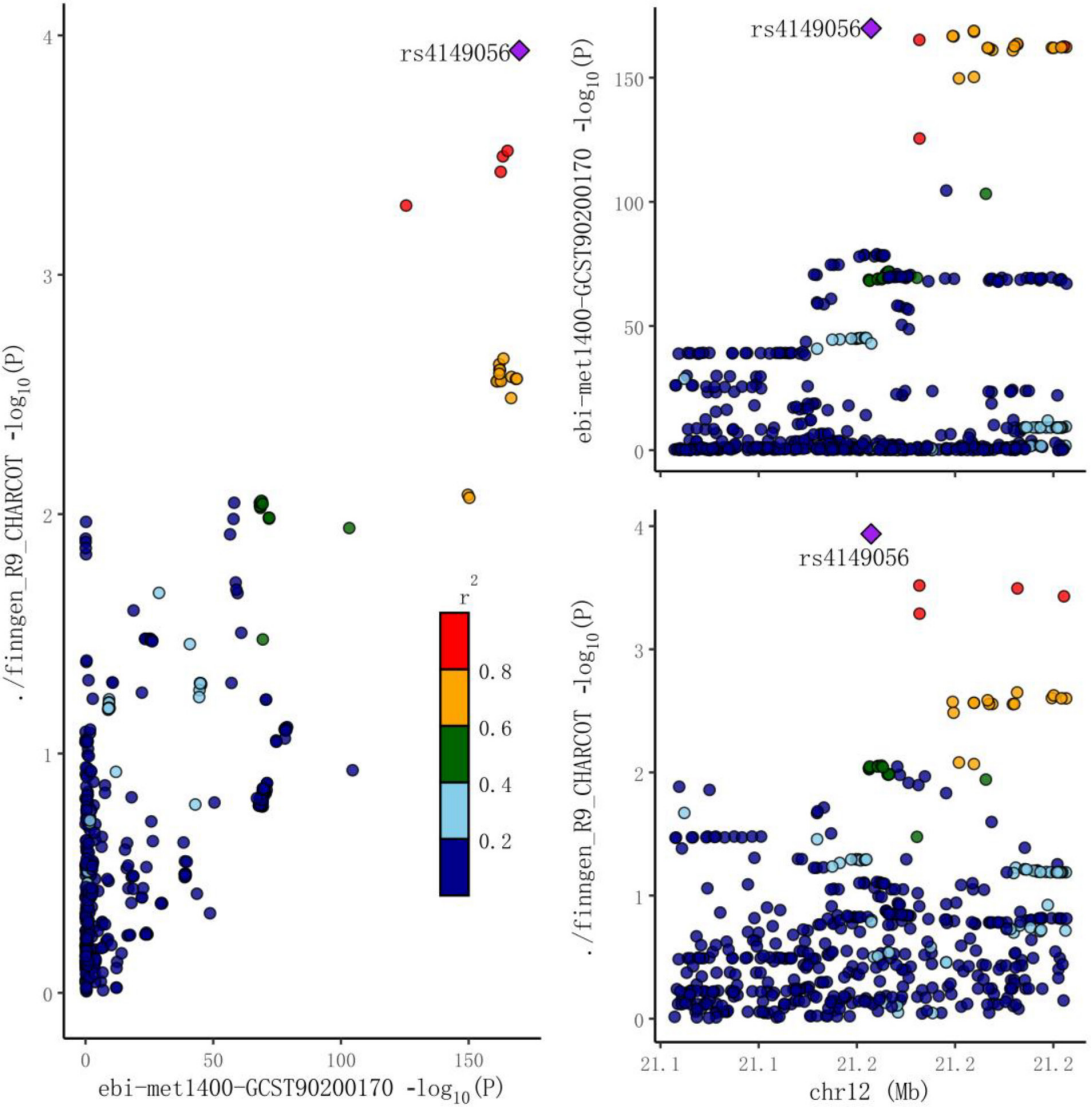
Colocalization at chr12 lead SNP rs4149056 between C18:2-DC and CF (PP.H4 = 0.945). Left: −log_10_P comparison, metabolite GWAS (GCST90200170) vs FinnGen CF; rs4149056 leads both datasets. Top right: regional plot for C18:2-DC. Bottom right: regional plot for CF. Color = LD *R*^2^ with rs4149056: red (> 0.8), orange (0.6–0.8), green (0.4–0.6), light blue (0.2–0.4), dark blue (< 0.2). Concordant signals support shared causal variant. C18:2-DC = octadecadienedioate, CF = Charcot foot, GWAS = genome-wide association study, LD = liquid disequilibrium, PP.H4 = posterior probability of H4, SNP = single-nucleotide polymorphism.

We conducted MVMR to assess whether the impact of C18:2-DC levels on CF was independent of common lifestyle factors and physical conditions, such as cigarette consumption, body mass index, and fasting glucose. After adjusting for these confounders, C18:2-DC levels remained negatively associated with CF (OR = 0.632, 95% CI 0.479–0.834, *P* < .05) (Table S8, Supplemental Digital Content 8). The link between C18:2-DC levels and CF was further validated by colocalization analysis (posterior probability of H4 = 0.945, Fig. 4, Table S9, Supplemental Digital Content 9), indicating shared causal variants between the 2 in the genomic regions surrounding the lead SNP, rs4149056.

## 4. Discussion

Utilizing large-scale GWAS data, we uncovered potential links between 60 metabolites and CF, with 34 displaying negative associations and 26 showing positive associations. Following adjustment of *P* using the Benjamini–Hochberg method (FDR < 0.05), we identified a significant negative impact of a unique metabolite, C18:2-DC, on CF (OR = 0.644, 95% CI 0.528–0.785, FDR = 0.018), a novel finding not previously reported. Furthermore, MVMR analyses substantiated the adverse association between C18:2-DC and CF (OR = 0.612, 95% CI 0.479–0.834, *P* < .05). Colocalization analysis confirmed our findings. These outcomes deepen our comprehension of the intricate association between metabolites and CF, potentially guiding innovative approaches for CF prevention and management. Furthermore, reverse MR analysis found no causal relationships from CF to any of the 1400 metabolites.

Several studies have consistently echoed our findings, demonstrating a link between plasma metabolite levels and the risk of CF. Jones et al examined a cohort of 2000 diabetes patients and discovered that certain lipid profiles were inversely associated with the occurrence of CF, further strengthening the validity of our results.^[32]^ Conversely, a particular case-control study failed to establish significant connections between plasma metabolites and the risk of CF.^[33]^ This discrepancy could potentially be attributed to variations in study design and smaller sample sizes in contrast to our study.

Inflammation has been pinpointed as a central element in the pathogenesis of CF.^[34]^

C18:2-DC, a long-chain dicarboxylic fatty acid, is an omega-oxidation metabolite of linoleic acid, an essential n-6 polyunsaturated fatty acid abundant in dietary vegetable oils.^[16]^ Structurally, C18:2-DC is characterized by an 18-carbon backbone with 2 carboxyl groups at both termini and 2 cis double bonds at positions 9 and 12, distinguishing it from monocarboxylic fatty acids and conferring unique metabolic properties. This metabolite is primarily generated through cytochrome P450-mediated omega-oxidation of linoleic acid in the liver, followed by peroxisomal and mitochondrial beta-oxidation.^[35]^ In our study, C18:2-DC demonstrated a significant negative association with CF risk (OR = 0.644, 95% CI 0.528–0.785, FDR = 0.018). The protective effect may be mediated through several plausible mechanisms. First, as a natural ligand for peroxisome proliferator-activated receptor gamma, C18:2-DC and related linoleic acid metabolites can suppress nuclear factor-kappa B activation and downregulate pro-inflammatory cytokines, including tumor necrosis factor-alpha, interleukin-1β, and interleukin-6.^[36]^ Second, C18:2-DC may modulate macrophage polarization, promoting the anti-inflammatory M2 phenotype while suppressing the pro-inflammatory M1 phenotype, thereby attenuating osteoclast-mediated bone resorption, a hallmark of CF pathophysiology.^[37]^ Third, dicarboxylic fatty acids have been shown to influence mitochondrial function and energy metabolism; under conditions of mitochondrial stress, elevated dicarboxylic acids may serve as alternative fuel sources, protecting cells from oxidative damage and apoptosis.^[38]^ In the context of diabetes, Zheng et al identified C18:2-DC as a significant mediator in the relationship between gut microbiota and type 2 diabetes, suggesting its involvement in metabolic homeostasis.^[39]^ Additionally, dicarboxylic fatty acids, including C18:2-DC, have been associated with reduced susceptibility to coronary heart disease and other inflammatory conditions, further supporting their anti-inflammatory and metabolic protective roles.^[40]^ The strength of the association observed in our study, confirmed across multiple MR methods and not solely driven by single IV results in LOO analysis, underscores the potential of C18:2-DC as a biomarker or therapeutic target for CF.

The MVMR analysis, which takes into account potential confounders such as lifestyle factors and physical conditions, further validates the independent negative association between C18:2-DC and CF. This suggests that the effect of C18:2-DC on CF is not muddled by these factors, bolstering the potential role of this metabolite in the pathogenesis of CF. The independence from confounding factors is imperative for establishing C18:2-DC as a viable candidate for future clinical applications.^[41]^ Moreover, colocalization analysis offers additional evidence for a shared genetic foundation between C18:2-DC and CF, particularly around the lead SNP rs4149056. This supports the idea that the observed association may be driven by shared pathogenic variants, providing valuable insights into the genetic architecture of CF and its metabolic correlates. The high posterior probability garnered in the colocalization analysis fortifies the argument for a shared etiology, which could guide the development of genetically informed prevention and treatment strategies.^[42]^

The role of the immune system in the pathogenesis of CF is not yet fully elucidated. However, the anti-inflammatory properties of C18:2-DC could potentially play a part. Metabolites such as C18:2-DC are recognized for their ability to modulate immune responses, and their dysregulation has been associated with various inflammatory conditions.^[43]^ Given that CF is marked by inflammation followed by bone and joint destruction, it is reasonable to hypothesize that C18:2-DC may help in reducing immune-mediated damage in CF.^[44]^ In addition, the colocalization analysis centered around the lead SNP rs4149056 provides genetic evidence of a shared causal variant between C18:2-DC and CF. This suggests a direct genetic link that could be crucial in deciphering the molecular mechanisms underlying CF. Such insight could lay the groundwork for pioneering therapeutic strategies that target metabolic pathways to prevent or manage CF.^[45]^ To translate our findings into clinical practice, several steps would be required to establish C18:2-DC as a viable biomarker. First, large-scale prospective cohort studies are needed to establish population-based reference ranges for plasma C18:2-DC levels, accounting for age, sex, body mass index, dietary intake, and metabolic status. Second, standardized analytical protocols using high-throughput mass spectrometry platforms should be developed and validated to ensure reproducible quantification across clinical laboratories. Third, case-control studies nested within diabetic populations would be essential to determine threshold concentrations below which CF risk is substantially elevated, thereby enabling risk stratification. Finally, interventional trials assessing whether dietary supplementation with linoleic acid or direct administration of C18:2-DC can modulate circulating levels and influence CF progression would provide critical evidence for its therapeutic potential.

Despite the compelling findings of our study, it’s crucial to recognize its limitations. Firstly, our research did not include wet-lab experiments, which could have provided additional validation for the observed associations between plasma metabolite levels and CF. Secondly, while the sample size was significant, it might still be deemed small for a condition as intricate as CF, potentially constraining the generalizability of our results. Additionally, our research is based on a European database, limiting the applicability of our conclusions to other ethnic groups. As we progress, it’s essential to confirm our findings through further research aimed at uncovering potential mechanisms. This could be accomplished by carrying out in vitro studies to examine the roles of these metabolites in cell lines or animal models. Thirdly, the absence of clinical validation analysis implies that the translational impact of our findings has not been fully assessed. Fourthly, as a TSMR study relying on publicly available GWAS summary statistics, we were unable to account for individual-level covariates such as age, sex, diabetes duration, or glycemic control status in our analyses. The metabolite GWAS was derived from a general population cohort with mixed diabetes status, while the CF GWAS was derived from a Finnish population-based cohort where all CF cases were diabetic patients. This discordance in diabetes status between the exposure and outcome cohorts is a limitation, as the genetic instruments for metabolite levels may operate differently in diabetic versus nondiabetic populations. Future studies with individual-level data, stratified GWAS analyses, or MR analyses restricted to diabetic populations are warranted to confirm our findings. Lastly, the employment of multiple datasets could introduce batch effects that might have influenced the results, despite our attempts to control for such variability.

## 5. Conclusions

Our study has identified noteworthy associations between plasma metabolite levels and the risk of CF using a MR framework. The consistency of these associations across various MR methodologies and the strong colocalization signals indicate a credible link that isn’t confounded by high LD. These findings offer fresh insights into the potential metabolic pathways implicated in the pathogenesis of CF and may set the stage for the creation of innovative diagnostic and therapeutic strategies. Incorporating our results with clinical trials and functional studies will be of paramount importance moving forward, with the ultimate goal of enhancing the management and outcomes for patients with CF.

## Author contributions

**Conceptualization:** Diya Xie.

**Data curation:** Lihang Yang, Cheng Li.

**Funding acquisition:** Fengmin Liu.

**Software:** Diya Xie.

**Supervision:** Fengmin Liu.

**Validation:** Daosen Zhou, Lihang Yang.

**Writing – original draft:** Daosen Zhou.

**Writing – review & editing:** Fengmin Liu.


















